# Stretching health diplomacy beyond ‘Global’ problem solving: Bringing the regional normative dimension in

**DOI:** 10.1177/1468018115599820

**Published:** 2015-12

**Authors:** Andrew F Cooper, Asif B Farooq

**Affiliations:** University of Waterloo, Canada; University of Waterloo, Canada; University of Toronto, Canada

**Keywords:** Global health governance, health diplomacy, health negotiation, regional health, World Health Organization

## Abstract

The importance of the regional dimension of health diplomacy is only gaining slow and uneven recognition. This is in many ways surprising. As demonstrated in the work of Deacon on the ‘globalization of social policy’, global social policy has been animated and debated not only at the multilateral level but at the regional level as well. But at least in the diplomatic literature, the importance of this regional dynamic (with a focus on diverse sites and actors and the pursuit of democratic control) has been missed. The objective of this article is to explore whether health diplomacy is catching up to this larger debate re-shaping the conceptualization and practice of diplomacy more generally. In some ways, the results may be counter-productive in that this shift may encourage an increasingly fragmented process. Yet, it may also point to some breakthroughs, with diplomats, acting as ‘go to’ personnel on the front lines of operational activity, enabling actors to integrate with one another to produce effective governance. In doing so, the regional dimension is given greater recognition as a component of health diplomacy, albeit in an uneven and sometimes awkward manner. Whereas global diplomacy generally emphasizes problem solving, the regional dimension is animated by a normative orientation.

## Introduction

The importance of the regional dimension of health diplomacy is only gaining slow and uneven recognition. This is in many ways surprising given the animated debate to a large extent triggered by academic work on the ‘globalization of social policy’ (see, for instance, Deacon, 2007, 2011; Yeates, 1999, 2002, 2007, 2014a, 2014b, 2014c). But in the diplomatic literature, the importance of regional sites of policy formulation, and regional conceptualizations and practice of health diplomacy, has lagged behind.

In some ways, health diplomacy is still highly ‘state centric’, states at the apex of the global system viewing health as an arena that is increasingly linked to national interests and the defense of sovereignty. This trend has been somehow reinforced by recent cases of pandemics as health became a matter of securing borders as much as societies. This view gave substance to the ‘securitization’ of health (Rushton, 2010), with issues such as severe acute respiratory syndrome (SARS) or Avian influenza being treated as threats to national security. The state-centric approach seemed resilient to the increasing role and presence of civil society organizations (CSOs), such as Médecins Sans Frontières/Doctors without Borders (MSF) and other social organizations as well as philanthropies, operating autonomously on the front lines of epidemics, such as Ebola, compensating for measures and a focus beyond that of ‘protecting own borders’ (MSF, 2014). What this suggests is that although the hold of the state remains strong in some areas of health diplomacy, in other areas there are signs of adaptability and willingness to share responsibilities. Yet dynamics in health diplomacy are mainly driven by problem-solving and issue-specific forms of networking, cast as a guide to tactical solutions to (global) health problems.

In this article, we argue that global health increasingly exhibits a dynamic relationship between multitudes of actors that can be analyzed through a framework of ‘push–pull’ effect. In particular, we contend that the political practice of health diplomacy offers opportunities for non-state actors to set and implement policy agendas as well as regional organizations to advance new normative lines and problem-solving responses that not only have the potential to narrow North–South divisions but also to break with traditional methods and practice of diplomacy.

### The uneven expansion of sites and actors in health diplomacy

The core of the diplomatic process in global health governance is giving way to multi-level and multi-dimensional relations among the actors addressing crossborder issues, and new and more complex practices of diplomacy (Kickbusch, 2013: 9–11). Although highly driven by what Robert Cox (1981) termed as problem solving, that is, driven by specific ways of tackling a (globalized) medical/epidemiological problem, the diplomatic camp is no longer a privilege of the traditional professional state diplomats representing government foreign affairs departments. The late 20th century witnessed the rising awareness of, and cluster of, new trans-border issues that transformed the practice of diplomacy in international relations. Furthermore, the state-centric focus proved to be limited in addressing such issues, opening the way to innovative normative and practical grounds redefining opportunities to think and practice health governance in ‘21st century diplomacy’.

We argue that the underlying driving force for such systemic shifts in international relations was the ‘sticky’ nature of the issues themselves. Many CSOs, for example, brought public attention onto many emerging critical social issues for policy change well before a state policy response was initiated in addressing them. Most notably, community-level action prompted the exigency of a national response for HIV/AIDS issue well before any official state-level response was initiated in Africa (The Joint United Nations Programme on HIV/AIDS [UNAIDS], 1997).

Thus, the rapid emergence of non-state actors in addressing critical social issues became a watershed for building a comprehensive ecology of actors and elements on which such issues still sustain, expand, and evolve. This is when an issue becomes ‘sticky’, with calls for more than ad hoc responses, in the order of long-term solutions with ample space for multiple actors, and institutional norms and regime complexes (Raustiala and Victor, 2004: 279). Sticky issues demonstrate that states do not have exclusive ‘ownership’ of an issue in the 21st century. Sticky issues have its own ‘self’, autonomous of how a country’s foreign policy is shaped. It involves a multitude of actors in the process of its governance and diplomacy. In other words, an issue is the content of diplomacy; context is the event that animates the issues into life; and multitude actors are the agents that fragment global governance. What it implies is that certain issues due to their complex and ongoing nature serve as a catalyst for a focused, albeit at times divergent, set of global responses.

**Figure 1.**
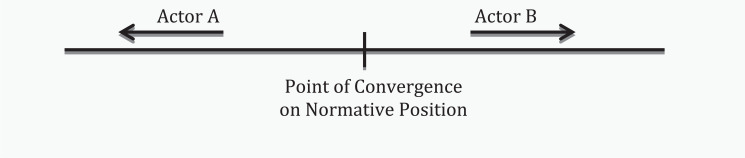
The ‘Push’ effect between actors.

Still, there continue to be elements of resistance from the traditional state authorities to address the emerging agenda and to adapt to changes that an effective solution – sometimes radical in nature – in response may call for. As a result, issue-specific CSOs may be in direct conflict with traditional state authorities. Indeed the contrast in normative positions acts to ‘push’ state and non-state actors further away from each other (see Figure 1). The ‘push’ effect is a natural tendency for the diverging actors in claiming the opposite pole to differentiate themselves notably on the question of the extent to which sovereign rights are defended or bypassed.

Fragmentation on health diplomacy is caused by the emergence of a multitude of actors. Actors, whether they are states, international governmental organizations (IGOs), international non-governmental organizations (INGOs), international organization (IOs), or CSOs emerge only when they acquire identities, which are shaped by and founded on their interests, functions, and policy around an issue. In terms of further analysis at the micro-level, the ideational affiliation of actors in international relations prior to their ‘emergence’ was ‘there’ but was not ontologically observable. Only when they became ‘fleshed out’ with missions, objectives, and modes of organizations did they effectively emerge as an actor. However, this type of actor emerges only *in relation* to other actors, mobilizes resources with different missions and objectives *compared to* that of others, and tries to accomplish something *in relation* to others.

**Figure 2.**
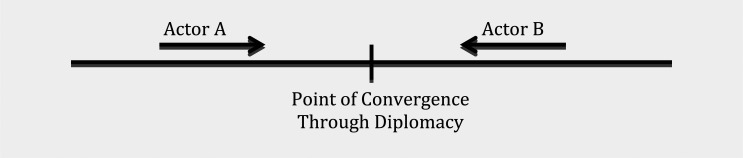
The ‘Pull’ effect between actors with/without a catalyst actor.

**Figure 3.**
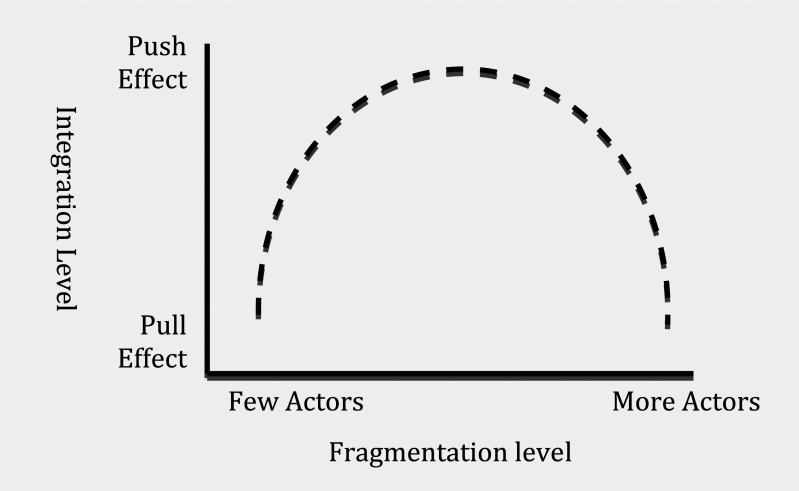
The inverted-U effect of the result of modern diplomacy.

Therefore, in functional terms, although divergence in issue-interest ‘pushes’ actors into opposite poles, there is a tendency among actors to ‘pull’ each other together in achieving a negotiated result because of the exigency of an issue (see Figure 2). Modern diplomacy is that ‘pull’ effect that builds partnership through negotiation, dialogues, and cooperation among a diversity of actors. The ‘pull’ effect in diplomacy, in effect, integrates fragmented international relations through a network of governance. As a result, the push–pull effect creates an inverted-U effect of this 21st century diplomacy in an increasingly fragmented world (Figure 3). In terms of timing, the pull effect may be a matter of a few years in some cases or a few decades in others. The pull effect between actors may result in a weak or a strong connection depending on how successful the negotiation is. Sometimes other actors act as a catalyst in initiating that pull effect which is in effect dialogue and negotiation between the diverging actors. IGOs often play that role when the IGO leaders press for a resolution to address an issue among its member states.

The nodes in the network connecting a multitude of actors with each other are effectively a form of communication, which could be via informal dialogues or formal negotiations. The diplomats are one of the many instrumental figures who are the ‘go to’ persons or the intermediaries and maintain those nodes. The nodes were predominantly among the states until the late 20th century in international relations. As such, states were the only powerful actors, diplomats were state-officials with traditional rules and customary conducts, diplomacy was ‘club-like’ with strong statecraft characteristics, and the nodal connections were mostly hierarchical. In the 21st century world, though, all nodes not necessarily direct toward states, rather they are poly-lateral; states are not the only powerful actors albeit still significant; diplomats are intermediaries who are not only state-officials; diplomacy is more than statecraft, with a mix of statecraft and society craft; and the nodal connections among the actors are more horizontal. The result is that ‘club diplomacy’ is supplemented by ‘network diplomacy’ (Heine, 2008: 272–277), where the institutional form for non-state actor is more resilient, horizontal information and resource mobilization are fluid, and clusters of networks are firmly ‘tied’ (Burris, Drahos and Shearing, 2005: 38) to nodes that act as a focal point for sticky issues.

Indeed, the emergence of unofficial (non-state) diplomats was a significant product of the process of fragmentation. The classic work of Hedley Bull (1977) did, to be sure, point to this phenomenon in its definition of diplomats by opening the way for a much broader categorization such that diplomacy not only includes the conduct of official relations by states but ‘other entities with standing in world politics’ (p. 162). Yet, as argued elsewhere, it took the structural and systemic contradistinction between the fragmenting global governance and the traditional diplomacy (Cooper et al., 2008: 2) to accelerate and diversify this process. In essence, forces on the ground caught up to conceptual possibilities, with the emergence of unofficial diplomats willing and able to fill in that ‘void’ created by the inadequacies of traditional diplomacy based on statecraft, which indeed is the embedded ‘pull’ effect of the fragmented international relations. Writing how diplomacy is changing in the presence of multiple actors in polycentric governance, Scholte (2008: 55–59) has argued that in the 21st century diplomacy, representation covers many more actors, communication handles faster speeds and larger volumes from multiple technological tools, and negotiation addresses an increasing range of diverse political parties and identities.

Amid this increasing fragmentation in the relocation of authority in the pattern of diplomacy (Kelley, 2010: 295), however, the regional dimension has received less attention. In part, this is due to both the perception and reality that regional organizations – or at least those in the global South – lacked problem-solving capacity. However, the gap is also a function of the contest between the problem-solving orientation of diplomacy at the apex of the global level and the dynamics at the regional level that highlight the need for the regional dimension as a driver for social redistribution and a set of expanded social regulations. Whereas the core of the problem-solving global diplomatic agenda has been heavily focused on pandemics, regional dynamics have concentrated on the extension of social rights. Indeed, even when there is an emphasis on lesson learning, the focus has often been on finding solutions involving equity (such as in the area of producing cheaper generic pharmaceuticals).

## The push–pull effect in global health diplomacy

In this section, we examine more closely how the 21st century of health diplomacy is shaped by a problem-solving bias shaped by a push–pull effect. In doing so, we illustrate how an instrumental as opposed to a normative lens differentiates the practice of diplomacy.

**Figure 4.**
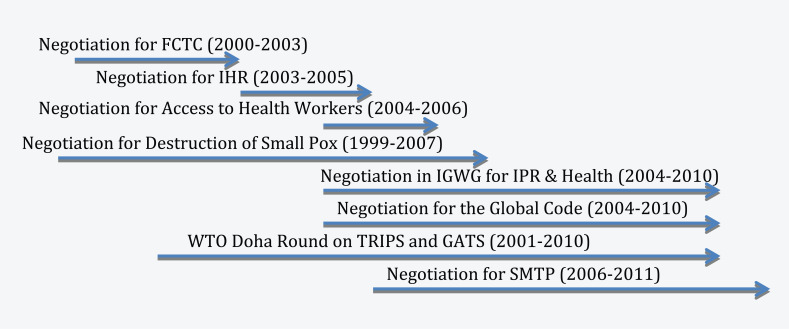
Timeline of recent health negotiations.

Health issues are still emerging and under-analyzed in the nexus between governance and diplomacy. Figure 4 shows that there have been multiple negotiations on critical global health issues in the last 10 years, which were triggered by the catalytic effect of a series of health emergencies. Therefore, global health governance is still at a formative stage and health diplomacy is very much in the transitional stage of practice in dealing, negotiating, and managing emerging health issues at different levels of governance (Lee and Smith, 2011; Novotny and Kevany, 2013). This process is constantly demonstrating that health diplomacy is more than about public health itself. Zucker (2012) wrote,

Public health negotiations are often about everything but public health. As such one much bring in the expertise from other domains including, international treaty law, finance ministries, trade delegations, foreign affairs, international security, and any other areas that are linked to the issues at hand. (pp. 273–274)

The pull effect in the practice of health diplomacy increasingly appears to be strong among the NGOs, networking with state and global and local actors. As stated by Low-Beer (2012), ‘a major trend since 2000 has been a shift to a wider “partnership approach” to delivering health aid, bringing together governments, private sector, civil society, private foundations and multilaterals, sometimes shouting and sometimes kicking’ (p. 23). Nevertheless, a multitude of actors also bring divergence in preferences and uncertainty in negotiation processes. Health diplomacy thus involves a multitude of inter-dependent actors, involved in poly-lateral diplomacy, and issues that create opportunities for partnership as well as conflict of interest.

Regional institutions in the Global South have been increasingly carving out a place in this problem-solving dynamic. For instance, African regional organizations such as South African Development Community (SADC) and the Economic Community of West African States (ECOWAS) have engaged with some limited success in addressing HIV/AIDS problem (Lisk, 2008: 160–161; Deacon et al., 2010 passim; see also Penfold and Fourie, this issue). The French non-governmental organization Agence de Médecine Préventive (AMP) focused on the issue of immunization in Africa and provided another excellent example of the multi-actor and multi-level interaction necessary to produce public goods for health at the regional as well as the national level. Utilizing the ‘Strengthening Independent Immunization and Vaccine Advisory Committees’ (SIVAC) initiative, AMP collaborated with the West African Health Organization (WAHO), the health branch of ECOWAS, to provide global recommendations to all countries in West Africa, especially with the establishment of National Immunization Technical Advisory Groups (NITAGs; Mengel et al., 2014). In South America, there is a long tradition of regional cooperation in health that can be traced to the establishment of the Pan-American Health Organization in 1902, but that is gaining new traction with the establishment of the Union of South American Nations in 2008 (see Herrero and Tussie, this issue; Riggirozzi, 2015).

Nonetheless, in the expansion of problem-solving mechanisms, there is a large gap between the activities at the global and regional level. NGOs and regional organizations are often at a disadvantage in terms of outreach and institutional capacity, as well as in terms of depth and experience in international law to effectively influence negotiation process in health-related issues (Taylor and Dhillon, 2011). For instance, health diplomacy is profoundly affected by legal considerations during negotiation process. Trade negotiations such as the General Agreement on Trade in Services (GATS) and Trade-Related Aspects of Intellectual Property Rights (TRIPS) include critical legal implications on generic drug production, public health services, price control of essential medicines, and market regulations. Indeed, the legal matters on trade affect multiple health negotiations simultaneously. During the negotiation on influenza virus sharing with Indonesia, France, the United Kingdom, and a few other countries indicated that the interdisciplinary working group (IDWG), formed by the World Health Organization (WHO), could not debate the intellectual property rights (IPR) issues as the WHO member states were waiting for a report from another negotiation on IPR (Lange, 2011: 138–139). Often changes in texts from ‘should’ to ‘shall’ stall last minute negotiations due to the legal implications associated with them, let alone the disagreements in legal niceties as evident from the Framework Convention on Tobacco Control (FCTC) negotiation (Bernard, 2012: 68).

Likewise, philanthropic foundations are another example of non-state actors playing a major role in global health, a trend that although not new has recently been intensified by activities of the Bill & Melinda Gates Foundation (Fidler, 2013: 701). The Gates Foundation is not only a larger health donor than all governments, except for the United States and the United Kingdom (McCoy et al., 2009), but it is also a crucial participant in negotiations across a wide spectrum from the ‘Health 8’ coordinating group to its 1.5 billion dollar contribution to the G8’s 2010 Muskoka Initiative on Maternal, Newborn, and Child Health (Fidler, 2013: 701). Significantly, the regional dimension is coming into focus in this domain. For example, in a meeting between the African Development Bank President Donald Kaberuka and Bill Gates, co-chair and trustee of the Bill & Melinda Gates Foundation, discussions were held on ways to cooperate on a range of issues from vaccine delivery to sanitation, to Ebola (African Development Bank Group, 2014). Constant efforts to improve epidemiological surveillance at the regional level are also evident from the earlier experience of the United States and the Public Health Agency of Canada (PHAC) helping setting up Polymerase Chain Reaction machines in Mexico for molecular diagnostics, signing a trilateral agreement of cooperation under the Security and Prosperity Partnership of North America, and more so after recent emerging disease outbreaks in Africa where a network of laboratories in Kenya and Ghana are key pillars of disease surveillance (Farooq and Majowicz, 2013: 40–41).

That being said, though, the normative orientation offers considerable compensation, as revealed by some earlier successful campaigns by the CSOs such as the boycott of Nestle on breast milk substitutes. The CSOs are also learning fast by sharing their global network of expertise. Indeed, South Africa’s Treatment Action Campaign’s (TAC) legal collaboration in 2001 is a perfect example (Berger, 2002; Heywood, 2009). TAC intervened as *amicus curiae* in the court proceedings between the South African government and the Pharmaceutical Manufacturers’ Association (PMA) when the former wanted to regulate drug pricing for better access to healthcare. TAC was supported by a global and local network of CSOs with legal expertise and, as a result, won the case for South Africa.

The increasingly significant role of non-state organizations and regional actors, different forms of relationships to develop, and inter-agency coordination and cooperation make health diplomacy more nuanced. However, there are structural disadvantages in terms of policy negotiation addressing global health issues. Their main tool remains the normative approach, and the issue-specific context, as demonstrated by the move by the WHO and its member states to negotiate the revised International Health Regulations (IHR, 2005). During the intense negotiation with Indonesia, the parallel outbreak of H5N1 helped WHO Director-General Margaret Chan to appeal to the parties of negotiation for swift compromise to restart virus sample sharing (Lange, 2011: 141).

Also the divergence between the North and the South is driven by distribution of power that is grounded on economic and institutional capacity. Power differentials between the developed and developing countries is historical that goes back to the colonial period. As a result, the low-income countries often negotiate as a bloc against the developed countries, which, however, is not unique to health diplomacy. Nevertheless, the ‘right to health’ provision, public health emergency due to AIDS, malaria, tuberculosis, and other diseases in developing countries, and high price of life-saving drugs and vaccines show the developing countries have vested interest to strongly contest negotiations with the developed countries who have the technological advantage, financial resources, and host the big pharmaceutical firms with preferential access to drugs and vaccine production during public health emergency.^1^

Under the GATS terms, market access liberalization restricts developing countries from providing critical health services through public sector, providing incentives to domestic firms over foreign companies, regulating the health sector, and managing the cost of essential health services (Mukhtar, 2012: 31–33). The Trade-Related Aspects of Intellectual Property Rights (TRIPS) also is a major concern for the developing countries because pharmaceutical patent would prevent generic drug production, which is vital to address various epidemics in developing countries. Therefore, during negotiation on these issues, developing countries ‘generally act together as one block, assigning a delegate from one country in the block to speak on behalf of all countries in the block’ (Mukhtar, 2012: 35).^2^ During the IHR negotiation process, disagreement on the Annex 2 of the final text of IHR (2005) clearly demonstrated divergence between the North and the South, where the developing countries argued that unless a list of diseases were specified in the IHR texts, they would be unable to get necessary financial support for disease surveillance (Katz and Muldoon, 2012: 92).

The pull effect is strong among the developing countries on many cases due to their common interests. The developing countries supported and sponsored Indonesia’s resolution when the latter stopped sharing the influenza viruses with the WHO Collaborating Centers in 2006 (Lange, 2011: 130–134). Indonesia had borne the brunt of avian influenza (AI) in that it experienced the highest number of AI human infections, with a staggering case-fatality rate of over 80%. Nonetheless, Indonesia’s refusal to share virus samples from new infections with the WHO’s Collaborating Centers marked a major ramping up in terms of resistance. For over 50 years, the Centers had served as a mainstay of international influenza surveillance, with the samples being used to facilitate vaccine production. Indonesia’s actions are widely interpreted by developed countries as being in clear violation of the revised IHR that came into effect in July 2007.

During the negotiation process, which led to the landmark Standard Material Transfer Agreement (SMTA) for equitable access to influenza vaccines, a clear division was observed between the developing and developed countries where the former pressed for obligatory terms for benefit sharing (Fidler, 2013: 165). On a regional basis, Association of Southeast Asian Nations (ASEAN) placed the precept of sovereignty and non-interference over problem solving although the issue had clear trans-border implications (Kamradt-Scott and Yoon, 2013: 103).

More generally, a strong division between North and South also complicated the negotiation for the destruction of the Smallpox virus stocks where India took the lead for the G77 group in 1999 (Tucker, 2002, 2011). The rhetoric in negotiation was often detrimental to the negotiation. Zucker (2012) wrote,

At time even the greatest amount of tension was mixed with a sardonic levity. The delegate from Thailand said that he ‘might just be a small sparrow, but he was not afraid of the big eagle’. Clearly, the eagle being a symbol of America, was a reference to the United States’ position on intellectual property rights. (p. 263)

The pull effect weakens when a lack of institutional capacity within the developing countries acts as a functional problem. It challenges the South making the most efficient use of their resources in health diplomacy. Furthermore, institutional incapacity challenges the developing countries with transactional cost to implement negotiated agreements. Negotiation in health-related issues often requires a wide array of experts skilled in legal, scientific, political, trade, and public health topics. Health diplomacy, which often includes arduous, long, and expensive negotiation process, spanning multiple years and involving multiple simultaneous negotiations, is a disadvantage for the developing countries that do not have enough financial and human resources to address domestic health problems, let alone the ability to send enough skilled officials with a wide array of expertise in all international negotiations.

Furthermore, sometimes the negotiation framework itself is a disadvantage for the developing countries working as a collective group such as in the World Trade Organization (WTO) negotiation on GATS and TRIPS. Lack of intra-agency coordination and cooperation in developing countries weakens their collective negotiation position and, in effect, weakens the pull effect among them (Mukhtar, 2012: 38–39). The inter-agency coordination and cooperation by the United States during the FCTC negotiation demonstrate the huge gap in such capacity between the North and the South (Bernard, 2012: 53–54). Lobbies for trade interest are also stronger which often put the health issues in the backbench, weakening a collective action by the developing countries (Mukhtar, 2012: 41). Although the African states championed in initiating the negotiation process for the WHO Global Code of Practice on the International Recruitment of Health Personnel, they could not engage in the negotiation until Norway and WHO EURO supported the Global Policy Advisory Council in ensuring their active participation (Taylor and Dhillon, 2011). Often the developed countries bear transactional costs – for equity – of developing countries to enable them to govern. Therefore, the developed countries too have vested interest for soft-binding negotiated outcomes to avoid bearing transactional costs for other countries.

Although the ‘push’ effect is strong between the developing and developed countries on many issues, security and sovereignty considerations often trump the North–South division. As a result, the push–pull effect *among* the developing countries is very dynamic too. The negotiation for the destruction of the Smallpox virus, which lasted from 1999 to 2007, illustrates the dynamic relations *between* the developed and developing countries and *among* the developing countries. When the world eradicated the Smallpox in the late 1970s, keeping the remaining stocks of the Variola virus appeared to be a security threat to global health. The developing countries from Africa and Asia with support from India, China, Iran, Cuba, and South Africa strongly opposed any delay in the destruction of the virus stockpile.

However, the United States was concerned about security threat from the undeclared stockpile by other countries such as Russia, China, Iraq, Iran, and Libya. India took the lead on behalf of the G77 developing group. India’s active negotiation with the United States aimed to lower the temporary retention timeline of the virus stockpile from an indefinite period to 2 years. However, during the course of the negotiation, India’s position drastically shifted after September 11 terrorist attack.The attack in the United States highlighted a potential security threat from bioterrorism. Russia, on the insistence of the United States, supported indefinite delay. China too followed suit at the end (see Tucker, 2006, 2009).

As noted above, another case of sovereignty question in global health diplomacy is Indonesia’s suspension of virus sharing with WHO’s influenza surveillance network in 2005. Indonesia claimed – although unsuccessfully – that pathogens were their sovereign right under the Rio Biodiversity convention (Vezzani, 2010). Similarly, during the negotiation of the IHR (2005), several states were not willing to discontinue certain national public health measures that were not supported by scientific studies. Katz and Muldoon (2012) wrote, ‘The states viewed this as an infringement on their sovereignty because it would prevent them from exercising public health authority within their own borders’ (p. 89). Other points that were debated during the negotiation on the ground of sovereign right included WHO’s surveillance system that depends on third-party sources, definition of disease, border control, and inclusion of chemical, radiological, biological, and nuclear events in the revised IHR draft (Katz and Muldoon, 2012: 88–91).

Similarly, the United States created numerous objections during the negotiation process of the FCTC drafting on the ground of their constitutional rights. Bernard (2012) wrote,

In the Bush Administration’s strict interpretation of the United States Constitution, the federal government’s authority to agree to treaty commitments is limited to the extent that it could not enter a treaty that imposed duties on a state or an of a state’s political subdivisions to take certain actions inconsistent with the powers reserved to the states by the Constitution. (p. 67)

Thus, compromise due to sovereignty and security concerns often results in soft-binding resolutions, leaving ambiguity in the text of negotiation, which provides states with flexibility to enforce at the national level.

Although sovereignty and security work as ‘push’ factors with a dynamic division in health diplomacy, common security interest often can work as a ‘pull’ effect as an incentive to cooperate with the international community. China withheld information during the SARS outbreak, which helped the spread of the virus and complicated the global response. However, China’s shift in policy after SARS outbreak embracing greater openness and multilateral cooperation also points to its realization that security benefit is higher in cooperation than withholding information in fear of economic loss (Schwartz and Schwartz, 2010). Success in health diplomacy is subject to a state’s security incentives to cooperate. However, states also like to keep reservation for their national security. Therefore, global security interest can effectively work as a ‘pull’ effect when soft-binding negotiated clauses or outcomes accommodate reservations for national interest.

As showcased throughout this special issue, there is some strong evidence that regional actors are becoming more robust in animating new norms to improve health rights in international arenas engaging in new forms of regional diplomacy. Such a dynamic may accentuate the gap between the problem-solving approach favored by the core actors in the global system and, in so doing, reinforce the differences vis-a-vis the North and the Global South. The advantage, nonetheless, is that new avenues are opening up about the possibilities of health diplomacy as an agent of transformation in terms of social justice. In declaratory terms, the call for bringing the regional dimension has been building for a considerable length of time (see Yeates and Deacon, 2010, and ‘Introduction’ to this Special Issue). More recently, Yeates (2014b) points toward the possibility of ambitious regional mechanisms including regional social funds and regional social rights in supporting the implementation of the Social Protection Floor (SPF) Initiative.

Still in terms of practice, the expansion of regional organizations directly into health diplomacy has lagged behind other manifestations of diplomatic change. The Caribbean Community and Common Market (CARICOM), for instance, has developed some guidelines for regional capacity for issues such as human resource capacity and communicative diseases (Yeates and Deacon, 2010) though Yeates (2010, 2014b) argues that there are tensions in the region between approaches that privilege the production of health workers for ‘export’ and those that favor regional self-sufficiency. It may be misleading to suggest that the disconnect between the declaratory and the operational necessarily stymies momentum for the regional dimension in health diplomacy. Rather, it signals forcefully the salience of the contest between two types of diplomacy: the problem-solving diplomacy at the core of the approach favored by the actors at the apex of the global system, and the normatively driven approach advocated by a number of regional actors in the global South. The developments in health diplomacy with respect to the regional dimension reveal the salience of this normative contribution. In light of this, Yeates (2014b: 3) argues that many of these regional social policies have progressed faster as exhortative declarations of aims and principles rather than as binding regulatory or redistributive mechanisms. However, it is important to note the symbolic and practical uses of exhortative policy (such as Social Charters and other declarations of intent) in creating greater awareness of a range of common issues and a world of possibilities on a wider front. Exhortative policy may not have ‘teeth’ but it is nonetheless vital in contributing to the forging of transnational political alliances and political dynamics that may in turn stimulate more substantive forms of regional cooperation and action (Yeates, 2014a).

What is most stimulating about the normative component is that it extends the agenda into the sphere of social equity and social justice, a focus neglected by the problem-solving approach in health diplomacy. Again, in a number of cases, there is a disjunction between declaratory and operational practice. As noted in the contribution by Amaya, Rollet, and Kingah in this Special Issue, the Healthy ASEAN 2020 document (ASEAN, 2000) makes the claim that health should be at ‘the centre of development’, utilized as a tool to enhance the well-being and the livelihood of the peoples of ASEAN. But the diplomatic focus of ASEAN has been concentrated specifically on problem solving, with a strong emphasis on health as a tool for regional stability and security as underpinned by the creation of regional mechanisms of health cooperation such as the ASEAN + 3 Partnership laboratories (APL) and the ASEAN + 3 Field Epidemiology Training Network (FETN).

The most compelling terrain for the momentum toward a normative approach on health diplomacy, nonetheless, is emerging in the Americas, particularly in the Union of South American Nations (UNASUR). Rather than defining normative objectives with a focus on specific (vulnerable and excluded) populations, or populations in high-risk areas – an approach associated with regional formations in Southern Africa where the presence of donors and philanthropies is significant in terms of agenda setting, funding, and implementation of projects (see Penfold and Fourie, this issue) – UNASUR focuses on structural issues affecting health provision and health governance, that is, social determinants of health and health promotion, leadership, and capacity building (Herrero and Tussie, this issue, Riggirozzi, 2015).

## Conclusion

The objective of this article has been to find out whether the conceptualization and practice of diplomacy are being reshaped in the health domain. Whereas diplomacy traditionally has been framed in a state-centric manner with the privileging of sovereignty and the national interest, health diplomacy contests this approach. Both the normative and operational claims to dealing with health as embedded in a unique ‘structure’ of global health governance was strong, claims that were reinforced when CSOs got greater access to specific issue areas of the health agenda.

Yet, the extent of this transformational shift has been uneven and awkward by the ‘missing’ regional element. Although a good deal of activity was taking place in different regional contexts by a wide number of actors, this phenomenon has been largely overlooked in the diplomatic literature, though not in the Global Social Policy literature.

Notwithstanding the embedded culture of continuity, especially as noted by Fidler (2013) at times of crisis (p. 705), diplomacy has bent if not broken the traditional model of diplomacy. There has been a huge proliferation of actors with competing ‘interests’ and ‘ideas’, which increasingly fragment the structure of global health. Significantly, in terms of a variety of substantive issues, there has been a move to stickiness, with a distinct ecology. The response to the West African Ebola epidemic reinforces this trend.

While it has taken longer for the regional dimension to take on a higher profile, the impact of this shift away from a subordination of the regional to the ‘global’ has serious consequences. If problem solving is a component of regional health diplomacy, the dominant force has been normative in nature, with a variety of regional institutions determined to push an agenda not only for order and stability but for rights and justice.

The danger, to be sure, is that this process toward competing actors with principles, agendas, and institutional norms will accentuate fragmentation further in health diplomacy. Yet this differentiated landscape, and contested values, also brings with it opportunities for creative debates and policy outcomes. In such a setting, the definition of the purpose of diplomacy is in flux. If mediation, negotiation, and representation have been the traditional focal points in the practice of a modern diplomacy, then this article identifies the need for building in an increasingly diverse context and content. Health diplomacy will continue to focus on outcomes, but the means and types of those instrumental deliverables will be much more variegated to incorporate changing regional realities.

## Notes

1. Del Rio and Hernandez-Avila (2009: 679) highlights the unequal distribution of vaccines during the pandemic in 2009.

2. In fact, one of the reasons that drove the developing countries band together on General Agreement on Trade in Services (GATS) and Trade-Related Aspects of Intellectual Property Rights (TRIPS) negotiation is that the earlier negotiation during the Uruguay round was not beneficial to the developing countries.
